# Covalent Grafting of Inorganic Selenium to the Water-Soluble and Nondigestive Chinese Yam Polysaccharides Causes Greater Protection of IEC-6 Cells with Acrylamide Injury

**DOI:** 10.3390/foods14091560

**Published:** 2025-04-29

**Authors:** Zhen-Xing Wang, Li-Li Zhang, Xin-Huai Zhao

**Affiliations:** 1 Key Laboratory of Dairy Science, Ministry of Education, Northeast Agricultural University, Harbin 150030, China; wangzxfood@126.com (Z.-X.W.); lilizhang@neau.edu.cn (L.-L.Z.); 2 School of Biology and Food Engineering, Guangdong University of Petrochemical Technology, Maoming 525000, China

**Keywords:** yam polysaccharides, selenylation, acrylamide, IEC-6 cells, toxicity, barrier function, MAPK signaling pathway

## Abstract

Acrylamide, a harmful substance generated during the normal thermal treatment of foods, has been shown to adversely affect human health, particularly the vital intestinal barrier function. Meanwhile, natural polysaccharides are recognized to exert an important biofunction in the intestine by protecting barrier integrity. In this study, the non-starch, water-soluble, and nondigestive yam polysaccharide (YP) was extracted from fresh Chinese yam, while two selenylated derivatives with different extents of selenylation were prepared via the HNO_3_-Na_2_SeO_3_ reaction system, and designated as YPSe-I and YPSe-II, respectively. Their protective activities and the associated molecular mechanisms of these substances against acrylamide-induced damage in rat intestinal epithelial (IEC-6) cells were thereby investigated. The experimental results demonstrated that the selenium contents of YPSe-I and YPSe-II were 0.80 and 1.48 g/kg, respectively, whereas that of the original YP was merely 0.04 g/kg. In IEC-6 cells, in comparison with YP, both YPSe-I and YPSe-II showed higher efficacy than YP in alleviating acrylamide-induced cell toxicity through promoting cell viability, suppressing the release of lactate dehydrogenase, and decreasing the generation of intracellular reactive oxygen species. Both YPSe-I and YPSe-II could also manifest higher effectiveness than YP in maintaining cell barrier integrity against the acrylamide-induced barrier disruption. The mentioned barrier protection was achieved by increasing transepithelial electrical resistance, reducing paracellular permeability, facilitating the distribution and expression of F-actin between the cells, and up-regulating the production of three tight junctions, namely ZO-1, occludin, and claudin-1. Additionally, acrylamide was observed to trigger the activation of the MAPK signaling pathway, thereby leading to cell barrier dysfunction. In contrast, YPSe-I and particularly YPSe-II were capable of down-regulating two MAPK-related proteins, namely p-p38 and p-JNK, and thereby inhibiting the acrylamide-induced activation of the MAPK signaling pathway. Moreover, YPSe-II in the cells was consistently shown to provide greater barrier protection than YPSe-I. In conclusion, chemical selenylation of YP could cause higher activity in mitigating acrylamide-induced cytotoxicity and intestinal barrier dysfunction, while the efficacy of activity enhancement was positively affected by the selenylation extent.

## 1. Introduction

Acrylamide, a hazardous contaminant in processed foods which have received thermal treatment, is primarily formed via the Maillard reaction between the reducing sugars (such as glucose, fructose, and others) and certain amino acids like aspartic acid [1]. Processed foods therefore serve as the primary sources of acrylamide. It is noteworthy that acrylamide can be absorbed by the human body through multiple pathways, including both the digestive and respiratory tracts, and skin and mucous membranes. Acrylamide in the body might lead to several adverse events. Once acrylamide enters the body, it is metabolized into the active product, namely glycidamide, under the action of cytochrome P450. Glycidamide exhibits a greater tendency to bind to the guanine in DNA, potentially inducing genetic mutations and other hereditary damage [2]. In addition, acrylamide and glycidamide can bind into hemoglobin and thus accumulate in tissues like the nervous system, liver, kidneys, and reproductive system [3]. Therefore, it is impossible to ignore the harmful effect of acrylamide on human health. A previous study demonstrated that acrylamide could inhibit the proliferation of intestinal epithelial cells, lead to intestinal inflammation, and inhibit the expression of tight junction (TJ)-related proteins via well-known oxidative stress, thereby causing harmful damage to the intestinal barrier [4]. Clearly, prevention of acrylamide-induced barrier damage to the intestine is vital for gut health. Fortunately, one dietary component, namely polysaccharides (particularly plant polysaccharides), is regarded as playing a positive role in safeguarding the intestinal barrier.

Yam (*Dioscorea opposite* Thunb.), a plant belonging to the *Dioscoreaceae* family, is cultivated globally in warmer regions for its edible tubers. Numerous studies have demonstrated that the major bioactive substances in yam are yam polysaccharides (YP) [5]. The monosaccharide composition and structure of YP are highly complex, predominantly existing in the form of hetero-polysaccharides [6]. The bioactivity of polysaccharides is considered to be closely related to their structural characters, encompassing the types of monosaccharides, molecular weights, and types of glycosidic bonds. Certainly, chemical modification of polysaccharides can alter the structure and bioactivity of native polysaccharides [7]. Se is present in the human body as one of the necessary trace elements. Notably, Se can augment the activities of these important selenium-containing enzymes (e.g., thioredoxin reductase and glutathione peroxidase), and therefore possesses an ability to prevent free radicals from inflicting harmful damage on both cells and tissues [8]. An insufficient intake of dietary Se can lead to serious health risks, including various cancers such as gastrointestinal cancer and prostate cancer, as well as cardiovascular diseases and diabetes [9]. Globally, levels of Se in soil are typically deficient, leading to inadequate dietary intake [10]. Thus, Se supplements (particularly Se-enriched food supplements) prove beneficial to individuals residing in these regions. The chemical forms of Se can be classified into two types: inorganic Se and organic Se. The absorption proportion of organic Se within the human body can exceed 80%, and it exhibits stronger bioactivity and relatively lower toxicity [11]. In contrast, inorganic Se has lower bioavailability but higher toxicity or side-effects in the body. Generally, naturally occurring organic Se substances mainly include selenylated polysaccharides, selenylated proteins, and selenylated amino acids. Employing chemical selenylation, inorganic Se groups (e.g., selenite) can be covalently grafted onto polysaccharides or proteins, yielding selenylated protein/polysaccharide derivatives with higher Se content and bioactivity. For example, longan polysaccharides with a covalent selenylation exhibited an increase in Se content and anticancer capacity in human colon cancer HT 29 cells [12], while selenylated YP also possessed higher antioxidant potential and greater ability to regulate glucose metabolism [13]. However, whether a covalent selenylation or Se-grafting of YP could bring about higher activity in the intestine to alleviate acrylamide-induced cytotoxicity and barrier dysfunction is still unsolved. Thus, such a study is necessary.

This research involved extraction of the water-soluble, nondigestible yam polysaccharides (YP) from fresh yam tubers, while YP was then modified through covalent selenylation by using a reaction system containing HNO_3_ and Na_2_SeO_3_ to generate two Se-grafted YP derivatives, namely YPSe-I and YPSe-II, which were different in Se content or selenylation extent. Utilizing YP as a control sample and rat intestinal epithelial (IEC-6) cells as model cells, a series of indicators of IEC-6 cells (with or without acrylamide exposure) were analyzed. These indicators included cell viability, lactate dehydrogenase (LDH) release, reactive oxygen species (ROS) production, transepithelial electrical resistance (TEER) value, and paracellular permeability. Simultaneously, the intercellular distribution and expression of F-actin, the expression of three genes and proteins associated with tight junction (TJ) proteins (i.e., ZO-1, occludin, and claudin-1), as well as the expressions of p-p38 and p-JNK involved in the MAPK signaling pathway, were also examined in the cells. The present investigation aimed to determine whether Se-grafting affects the ability of YP in the intestinal environment to protect the intestine against acrylamide-induced cytotoxicity and barrier dysfunction, and to verify whether Se-grafting is a potential approach to enhance the bioactivity of natural polysaccharides.

## 2. Materials and Methods

### 2.1. Chemicals and Reagents

The fresh yam tubers were purchased commercially from the local market in Harbin (Heilongjiang Province, China). Key reagents, including acrylamide (CAS No. 79-06-1), fluorescein sodium (FS-Na), 4 kDa fluorescein isothiocyanate-dextran (FD-4), 3-(4,5-dimethyl-2-thiazolyl)-2,5-diphenyl tetrazolium bromide (MTT), and Dulbecco’s modified Eagle’s medium (DMEM) were procured from Sigma-Aldrich Co. in St. Louis, MO, USA. Additional materials such as phosphate-buffered saline (PBS) and dimethyl sulfoxide (DMSO) and were obtained from Solarbio Science and Technology Co., Ltd. in Beijing, China, whereas alkaline protease was purchased from Beijing Aoboxing Biotechnologies, Inc. (Beijing, China). All other chemicals employed in this investigation were of analytical grade, and ultrapure water was produced using a Milli-Q Plus system from Milipore Corporation in New York, NY, USA.

Actin-Tracker Red-Rhodamine, phenylmethanesulfonylfluoride (PMSF), bicinchoninic acid (BCA), and radioimmunoprecipitation assay (RIPA) kits were acquired from Beyotime Institute of Biotechnology in Shanghai, China. For RNA extraction, the RNAprep Pure Cell Bacteria Kit came from Tiangen Biotech in Beijing, China, while the NovoScript^®^ Two-Step RT-PCR Kit and SYBR qPCR SuperMix Plus were obtained from Novoprotein Biotech in Suzhou, China. Primary antibodies, including GAPDH (Bioss bs-10900R), ZO-1 (Bioss bs-1329R), and the goat anti-rabbit secondary antibody were purchased from Bioss Biotechnology in Beijing, China. Additionally, occludin (13409-1-AP) and claudin-1 (28674-1-AP) were sourced from Proteintech Group in Wuhan, China. The phospho-specific antibodies p-p38 (#4511) and p-JNK (#4668) were procured from Cell Signaling Technology in Danvers, MA, USA.

### 2.2. Cell Line and Cell Culture

IEC-6 cells sourced from the esteemed American Type Culture Collection (Rockville, MD, USA) were employed as model cells in this study. These cells were nurtured in a specialized DMEM medium, bolstered with a blend of antibiotics and growth factors. Penicillin and streptomycin were included at a strength of 100 U/mL, while fetal bovine serum at 10%, bovine insulin at 0.1% units/mL, and sodium pyruvate at 1% were used. The cellular habitat was carefully regulated, maintaining a temperature of 37 °C in a controlled humidified environment with a 5% CO_2_ level.

### 2.3. Preparation and Chemical Selenylation of YP

As previously described [14], YP isolated from fresh yam tubers was freeze-dried after ethanol precipitation. Subsequently, 1 g of YP was subjected to Se-grafting using 5% (*v*/*v*) HNO_3_ and either 50 or 100 mg Na_2_SeO_3_ [15]. After performing the selenylation reaction and ethanol precipitation, three rounds of ethanol washing were conducted to remove the unreacted Se. Following the freeze-drying process, two covalently Se-grafted YP derivatives, namely YPSe-I and YPSe-II, were successfully synthesized and employed in further testing for this study alongside the unmodified YP as a control. Furthermore, the Se contents of three polysaccharide samples were routinely measured using an inductively coupled plasma-mass spectrometer from Agilent Technologies (Santa Clara, CA, USA).

### 2.4. Assay of Cell Viability

Routinely, the cells were distributed into 96-well plates at a fixed density of 2 × 10^3^ cells per well, and allowed to incubate for 24 h. Following this, they were subjected to a 12 h serum-free medium treatment. After being rinsed three times with 10 mmol/L PBS solution at pH 7.2, the cell viability was assessed using the standard MTT protocol [16]. Similarly, a microplate reader (Bio-Rad Laboratories, Hercules, CA, USA) was used to measure the optical density of the wells at a wavelength of 490 nm.

To evaluate acrylamide cytotoxicity, the cells were incubated as above. After cell washing with the PBS, the cells were then treated with acrylamide at 1.25, 2.5, 5, and 10 mmol/L. The cells underwent a further 24 h incubation period. The cells’ viability was measured using the MTT method. The cells treated with normal medium only were utilized as a control (set with viability value of 100%) [17]. The IC_50_ value of acrylamide was calculated according to a past study [18].

To evaluate the effect of YP, YPSe-I, and YPSe-II on the cells injured by acrylamide, the cells were incubated as described above. After the incubation, the supernatant was discarded. The cells were then treated with polysaccharide samples (dose levels ranging from 5 to 40 μg/mL) for two time periods (12 and 24 h), followed by a 24 h incubation with 2.5 mmol/L acrylamide. The supernatant was discarded; meanwhile, the cell viability was assessed as above. The viability value was then expressed as a percentage relative to the control cells (not exposed to acrylamide-induced damage) [19]. The control cells as usual were also designated with a cell viability value of 100%.

### 2.5. Assay of Transepithelial Electrical Resistance (TEER) Value

Amounts of 0.5 mL of the cells at a density of 2 × 10^5^ cells/mL were seeded into the Transwell inserts of 12-well plates (with a pore size of 0.4 μm), and 1.5 mL of the culture medium were added to the basolateral side. The culture medium was replaced every other day until the TEER value of the cell monolayer was 50 Ω cm^2^. TEER value was measured via a Millicell-ERS2 volt-ohmmeter (Millipore, Bedford, MA, USA). The cells were transferred to a serum-free culture medium and incubated for 12 h. Subsequently, 0.5 mL of the medium containing the polysaccharide samples (concentrations ranging from 5 to 40 μg/mL) were added to the apical side, and 1.5 mL of the culture medium were added into the basolateral side. The cells were cultured for 12 and 24 h, while liquid in the apical sides was replaced by 2.5 mmol/L acrylamide. Subsequently, the cultures were further incubated for another 24 h. TEER value was measured both before and after these treatments, and calculated according to a past study [20].

### 2.6. Paracellular Permeability Assay

The cells were introduced to the apical side of 12-well Transwell inserts (0.4 μm pores), following an identical treatment protocol to the TEER assay. After being treated with the polysaccharide samples at concentrations ranging from 5 to 40 μg/mL for 12 and 24 h, the cells were exposed to 2.5 mmol/L acrylamide for 24 h. Subsequently, 0.5 mL of FD-4 (0.5 mg/mL) or FS-Na (0.08 mg/mL) were added to the apical side of the Transwell plates. After a 24 h incubation period at 37 °C, 100 μL of liquid were drawn from the exterior of the basolateral side. The fluorescence intensity was then quantified using a TECAN Infinite M200 pro fluorescence microplate reader (Männedorf, Switzerland). The excitation/emission wavelengths to determine FD-4 and FS-Na were 490/520 nm and 430/540 nm, respectively [21,22]. The percentage value of the treated cells in comparison to the control cells (designated with paracellular permeability of 100%) was utilized to represent their paracellular permeability.

### 2.7. LDH Release Assay

IEC-6 cells were plated in 96-well plates at a cell concentration of 2 × 10^3^ cells per well and allowed to adhere for 24 h. Following this cell incubation, the medium was replaced with serum-free conditions for another incubation period of 12 h. The cells were exposed to varying concentrations of the polysaccharide samples (5–40 μg/mL) for either 12 or 24 h. Subsequently, 2.5 mmol/L acrylamide was introduced, and the cells were incubated for an additional 24 h. Finally, the cells were centrifuged at 190× *g* for 5 min. The supernatant was collected, and the value of LDH release was detected using the LDH detection kit. The cells treated solely with the normal medium were used as a control, and the LDH release value of the control cells was set at 100% [23].

### 2.8. Assay of ROS Production

IEC-6 cells were dispersed into 6-well plates, with each well receiving 4 × 10^5^ cells attached to the plate, and grown for 24 h. Following the cell attachment, the cells underwent serum starvation for 12 h in the serum-free medium. Subsequently, the culture medium was replaced by either fresh medium or the polysaccharide-containing medium (2 mL/well, 5–40 μg/mL concentration range) for an experimental treatment for either 12 or 24 h. Following acrylamide treatment (2.5 mmol/L, 24 h), a ROS fluorescent probe (2′,7′-dichlorodihydrofluorescein diacetate, at a concentration of 5 μmol/L) of 1 mL was utilized for cell staining in the dark for 20 min. The cells were then transferred to 96-well plates for fluorescence quantification. Measurements were performed using the microplate reader with excitation/emission wavelengths set at 488 nm and 525 nm, respectively. The results were reported as the relative ROS level in the cells, which was calculated as a percentage relative to that of the control cells. As usual, the relative ROS level of the control cells was set at 100% [24].

### 2.9. Observation of the Intercellular Distribution of F-Actin

Cell suspension (2 × 10^5^ cells, 1 mL) was plated in 12-well plates and incubated for 24 h, followed by serum starvation for 12 h. Subsequently, the cells were incubated with 40 μg/mL polysaccharide-containing medium for either 12 or 24 h prior to 24 h exposure to 2.5 mmol/L acrylamide. The untreated cells maintained in normal medium served as a control. After being washed twice with 0.1 mol/L PBS (pH 7.2), the cells were fixed with 4% paraformaldehyde in PBS for 15 min, and washed twice with PBS containing 0.1% Triton X-100. As per the F-actin kit protocol, the diluted working solution was added to the fixed cells, which were incubated for 1 h at 20 °C in the dark. Afterwards, the cells were washed twice with the supplemented PBS. The nuclei were stained with DAPI solution for 5 min, while the cells were washed three times with the PBS. Subsequently, F-actin distribution among the cells was observed and analyzed using a fluorescence microscope (OLYMPUS IX71, OLYMPUS Corporation, Tokyo, Japan) [25]. The cells cultured solely in normal medium were used as a control throughout the experiment.

### 2.10. Quantitative Real-Time PCR and Western Blot Analyses

IEC-6 cells were seeded into 6-well plates at 4 × 10^5^ cells per well. After 24 h incubation, the cells underwent 12 h of serum deprivation, followed by treatment with the three polysaccharide samples (40 μg/mL) for 24 h. Subsequently, 2.5 mmol/L acrylamide was applied to the cells for 24 h to induce cell injury. Total RNA was extracted from the cells in accordance with the protocol of the RNAprep kit, and subsequently reverse transcribed into cDNA via the PCR reverse transcription kit. PCR detection was performed using the GO Taq^®^ Qpcr Master Mix Kit and StepOnePlus real-time polymerase chain reaction system (Life Technologies Corp., Carlsbad, CA, USA). Glyceraldehyde-3-phosphate dehydrogenase (GAPDH) served as the housekeeping gene to normalize expression data, with claudin-1, ZO-1, occludin, p-JNK, and p-p38 designated as the target genes. All primer sequences (Table 1) were commercially designed and manufactured by Shanghai Sangon Bioengineering Co., Ltd., Shanghai, China. The gene expression levels were estimated using the 2^−ΔΔCt^ method [26].

The cells were seeded in 6-well plates (4 × 10^5^ cells/well). Following the treatment protocol used for RT-qPCR analysis, the cells underwent three PBS washes. Subsequently, 0.5 mL of RIPA cell lysis buffer supplemented with 1 mmol/L of the protease inhibitor PMSF of was added, following which the cells were routinely lysed on ice for 30 min. The cells were transferred to pre-cooled centrifuge tubes and then centrifuged at 14,000× *g* for 5 min. The supernatant, which represented the total protein solution, was collected thereafter. The total protein content was measured using a BAC kit. The same amount of total protein samples was denatured and then separated into 12% SDS-PAGE gels. Following electrophoresis, the gels were transferred onto nitrocellulose membranes that were then blocked with 5% skimmed milk powder in the TBST blocking buffer at 37 °C for 2 h. The membranes were washed three times with the TBST for 10 min each time. After being rinsed, the membranes were incubated overnight at 4 °C with the diluted working solution of the primary antibody (with a dilution factor of 1000). After being washed three times with the TBST, the membranes were incubated with the working solution of the secondary antibody (with a dilution factor of 5000) at 37 °C for 2 h, washed three times with TBST for 10 min each time, and then treated with ultrasensitive chemiluminescence solution. The protein bands on the membranes were thus visualized using a chemical imager (Fujifilm, Tokyo, Japan). Expression levels of the target protein were quantified using Image J software version 1.50b (NIH, Bethesda, MD, USA) [27], with GADPH as the internal reference.

### 2.11. Statistical Analysis

The reported data reflected the average results from three independent trials, and were expressed either as means or means ± standard deviations. Significant differences (*p* < 0.05) between different groups were assessed using one-way analysis of variance (ANOVA) with Duncan’s multiple range tests, which were conducted using SPSS 22.0 software (SPSS Inc., Chicago, IL, USA).

## 3. Results

### 3.1. Cytotoxic Effect of Acrylamide on IEC-6 Cells

In this study, the original YP contained only 0.04 g/kg of Se, but the two modified forms (YPSe-I and YPSe-II) showed substantially higher Se levels at 0.80 and 1.48 g/kg, respectively. The dramatic increase in Se content strongly indicates that selenite groups were successfully incorporated into the polysaccharide molecules via covalent grafting. Moreover, YPSe-II demonstrated a greater selenylation extent than YPSe-I. The elevated Se levels in YPSe-I and YPSe-II suggest that they might exhibit distinct biological activity in IEC-6 cells, compared to the original YP, because they had different Se contents.

**Figure 1:**
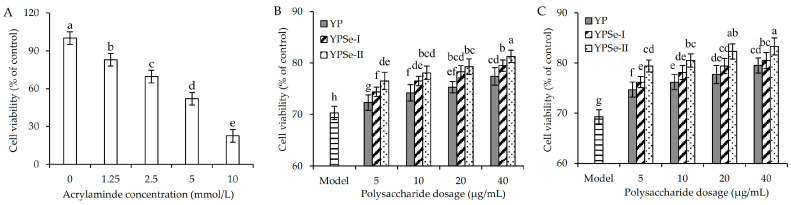
Acrylamide cytotoxicity in IEC-6 cells with a treatment time of 24 h (**A**), and viability of the acrylamide-injured IEC-6 cells treated with three polysaccharide samples (YP, YPSe-I, and YPSe-II) for 12 (**B**) and 24 h (**C**). Distinct lowercase letters above the labeled columns indicate that one-way ANOVA of the mean values differs significantly (*p* < 0.05).

An MTT assay was utilized to assess acrylamide-induced cytotoxicity in IEC-6 cells (Figure 1A). Upon exposing IEC-6 cells to acrylamide at 1.25–10 mmol/L doses for 24 h, the measured viability values showed a clear decrease. Viability values were reduced from 100% (the control cells) to 22.5–82.8%. Based on these measured viability values, the IC_50_ value of acrylamide was estimated to be 5.82 mmol/L. Acrylamide at a dose of less than 2.5 mmol/L showed a lower toxicity towards the cells, whereas a dose exceeding 2.5 mmol/L caused a clear cytotoxicity and led to cell injury (or lower cell viability). An acrylamide dose of 2.5 mmol/L was thereby selected for later experiments to induce injury in the cells.

To select the appropriate dose for the assessed polysaccharide samples, IEC-6 cells were also treated with the three polysaccharide samples at four doses for either 12 or 24 h prior to the acrylamide-induced injury. The results demonstrated that these samples exhibited higher viability values in a dose-dependent manner (Figure 1B,C), indicating that they could alleviate the cell damage induced by acrylamide. Generally, the ability of these samples to enhance cell viability was observed in an interesting order of YP < YPSe-I < YPSe-II. It was thus suggested that the employed covalent Se-grafting of YP and a higher selenylation extent caused higher activity in combating the acrylamide-induced cell cytotoxicity.

### 3.2. Effects of Three Polysaccharide Samples on LDH Release and ROS Level in Injured Cells

**Figure 2:**
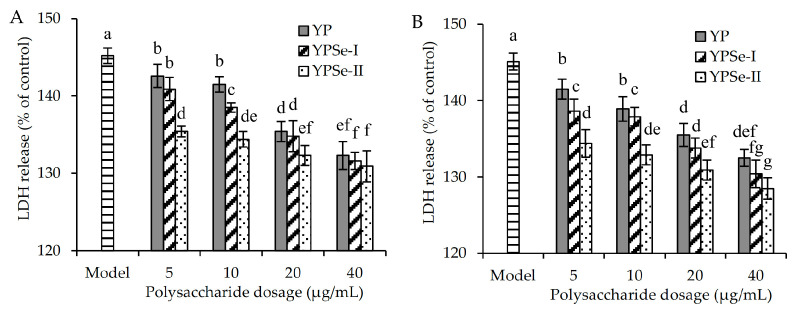
LDH release of acrylamide-injured IEC-6 cells treated with three polysaccharide samples (YP, YPSe-I, and YPSe-II) for 12 (**A**) and 24 h (**B**). Distinct lowercase letters above the labeled columns indicate that one-way ANOVA of the mean values differs significantly (*p* < 0.05).

LDH is a durable intracellular enzyme present within living cells. When the structure of the cell membrane is damaged or the cells undergo death, LDH will be promptly released from the cells. Thus, detection of LDH activity in the cell culture supernatant could reflect the extent of cell damage. In this study, amounts of LDH released by the cells treated with acrylamide in the presence or absence of YP, YPSe-I, and YPSe-II are shown in Figure 2. The model cells treated with 2.5 mmol/L acrylamide only had cell damage and therefore had a higher LDH release value than the control cells (145% vs.100%). When the cells were also treated with the polysaccharide samples for 12 h prior to the acrylamide injury, the respective values of LDH release were decreased in a dose-dependent manner to 132.3–142.6%, 131.6–140.9%, and 130.9–135.4%. If the polysaccharide samples were applied to the cells for 24 h, the values of LDH release were decreased to 132.5–141.5%, 130.4–138.5%, and 128.5–134.4%, respectively. The polysaccharide samples were able to suppress LDH release during cell injury. YP showed the lowest activity, while YPSe-II exhibited the highest. Se-grafting and a higher selenylation extent resulted in higher activity for the two selenylated derivatives in mitigating acrylamide-induced cellular damage.

**Figure 3:**
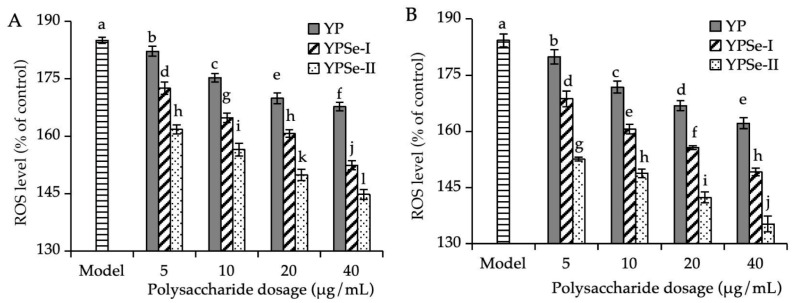
Relative ROS levels of acrylamide-injured IEC-6 cells treated with three polysaccharide samples (YP, YPSe-I, and YPSe-II) for 12 (**A**) and 24 h (**B**). Distinct lowercase letters above the labeled columns indicate that one-way ANOVA of the mean values differs significantly (*p* < 0.05).

In humans and animals, exposure to acrylamide can disrupt the balance of the redox system and mitochondrial respiration. Excessive ROS is thus generated. ROS generation in the studied IEC-6 cells is shown in Figure 3. Regarding ROS generation in the control cells, the model cells were considered to have much higher ROS production (184.3% vs. 100%). Acrylamide within the cells thus effectively promoted the generation of ROS and induced oxidative stress. However, when the cells were treated with YP (5–40 μg/mL) prior to the acrylamide injury, ROS production within the cells was reduced dose-dependently to 167.7–182.2% (12 h) or 162.2–179.9% (24 h). When the cells were exposed to YPSe-I or YPSe-II for 12 h, ROS production within the cells was decreased to 152.4–172.5% (or 144.8–161.8%). When IEC-6 cells were treated with the two Se-grafted derivatives for 24 h, ROS production was decreased to 149.2–168.7% (YPSe-I) or 135.3–152.6% (YPSe-II). Thus, YP, YPSe-I, and YPSe-II were all capable of alleviating the acrylamide-induced ROS generation and oxidative stress. Consistently, higher doses of the polysaccharide samples led to lower ROS production, while YPSe-II and YP exhibited the respective strongest and weakest activity within the cells. Consequently, this Se-grafting of YP and higher selenylation extent resulted in enhanced activity for the two Se-grafted products in mitigating acrylamide-induced oxidative stress.

### 3.3. Effects of Three Polysaccharide Samples on Barrier Integrity of Injured Cells

**Figure 4:**
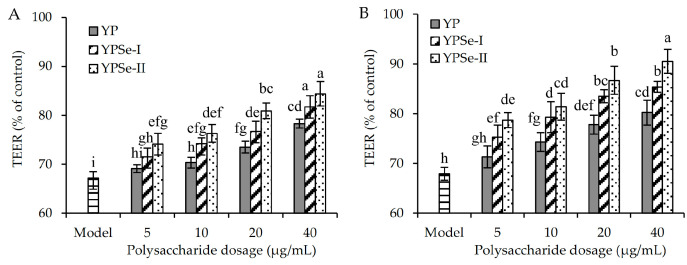
Transepithelial electrical resistance (TEER) values of acrylamide-injured IEC-6 cells treated with three polysaccharide samples (YP, YPSe-I, and YPSe-II) for 12 (**A**) and 24 h (**B**). Distinct lowercase letters above the labeled columns indicate that one-way ANOVA of the mean values differs significantly (*p* < 0.05).

For the purpose of revealing whether the polysaccharide samples could enhance the barrier integrity of IEC-6 cells upon acrylamide damage, the TEER values and paracellular permeability (cumulative permeation amount of FD-4 or FS-Na) of the cell monolayer under different treatments were measured and compared. The results showed that the model cells had a significant reduction in TEER value, compared to the control cells (67.2% versus 100%) (Figure 4). This indicated that acrylamide within the cells induced barrier loss. However, YP, YPSe-I, and YPSe-II could increase the TEER value of the treated cell monolayer. Specifically, the TEER values of the cells treated with the polysaccharide samples for 12 h were enhanced to 69.1–78.3%, 71.5–81.7%, and 74.1–84.4%, while those of the cells incubated with the polysaccharide samples for 24 h were increased to 71.3–80.2%, 75.3–85.4%, and 78.7–90.5%, respectively. The polysaccharide samples were therefore regarded as capable of restoring the barrier integrity of the cells. YP exhibited the lowest ability to rescue barrier integrity, whereas YPSe-II manifested the highest ability. Consequently, Se-grafting and higher selenylation levels endowed the two Se-grafted products with enhanced activity in restoring the acrylamide-induced barrier damage.

**Figure 5:**
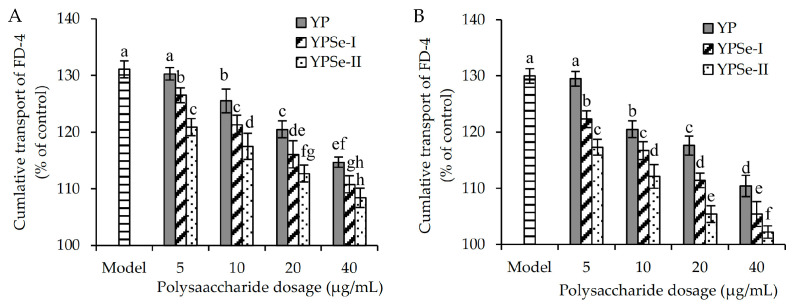
Cumulative transport for FD-4 of acrylamide-injured IEC-6 cells treated with three polysaccharide samples (YP, YPSe-I, and YPSe-II) for 12 (**A**) and 24 h (**B**). Distinct lowercase letters above the labeled columns indicate that one-way ANOVA of the mean values differs significantly (*p* < 0.05).

**Figure 6:**
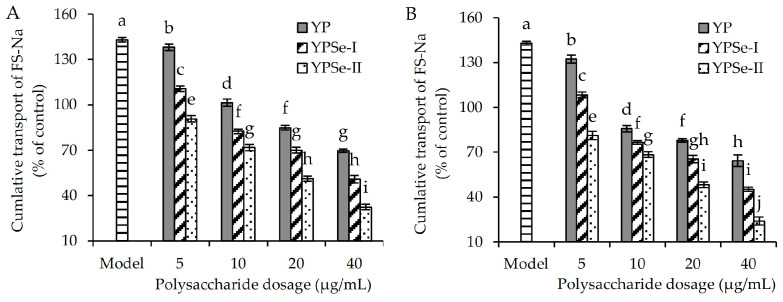
Cumulative transport for FS-Na of acrylamide-injured IEC-6 cells treated with three polysaccharide samples (YP, YPSe-I, and YPSe-II) for 12 (**A**) and 24 h (**B**). Distinct lowercase letters above the labeled columns indicate that one-way ANOVA of the mean values differs significantly (*p* < 0.05).

YP, YPSe-I, and YPSe-II, also in a dose-dependent manner, were able to decrease the paracellular permeability of the assessed cell monolayer. Taking the cumulative transmittance of FD-4 as an indicator and using as control cells those with 100% cumulative transmittance of FD-4 (Figure 5), it was observed that the model cells had highly increased cumulative transmittance of FD-4 (130.0–131.1%). When the polysaccharides samples were used to incubate the cells for 12 h, the detected values of the cumulative transmittance of FD-4 were reduced to 114.7–130.2%, 110.8–126.5%, and 108.4–120.9%, respectively (Figure 5). When the three polysaccharide samples were used to incubate the cells for 24 h, the measured values of the cumulative transmittance of FD-4 were reduced further to 110.4–129.5%, 105.4–122.3%, and 102.2–117.2%, respectively (Figure 5). Taking the cumulative transmittance of FS-Na as another index and using as control cells those with 100% cumulative transmittance of FS-Na, the model cells were also found to have much higher cumulative transmittance of FS-Na than the control cells (143.0% versus 100%) (Figure 6). Upon treating the cells with YP, YPSe-I, and YPSe-II at each of the four dose levels for the time durations of 12 and 24 h, the measured values of the cumulative transmittance of FS-Na were decreased to 69.6–138.1%, 50.8–110.7%, and 32.5–90.8%, respectively (Figure 6). When the three polysaccharide samples were used to incubate the cells for 24 h, the detected values of the cumulative transmittance of FS-Na were then decreased to 64.3–132.3%, 45.2–108.4%, and 23.9–81.2%, respectively (Figure 5). We suggest that the three polysaccharide samples restored the injured barrier function via reducing the enlarged paracellular permeability.

In total, the three investigated polysaccharide samples could rejuvenate the barrier integrity of the injured IEC-6 cells by elevating their TEER value and lowering paracellular permeability. Data comparison revealed that YP and YPSe-II exhibited the minimum and maximum activities within the cells, respectively. It was thus concluded that both covalent Se-grafting and higher selenylation extent caused higher activity of the two Se-grafted derivatives (i.e., YPSe-I and YPSe-II) in maintaining the studied barrier integrity of IEC-6 cells.

### 3.4. Effects of Three Polysaccharide Samples on F-Actin Distribution Among IEC-6 Cells

**Figure 7:**
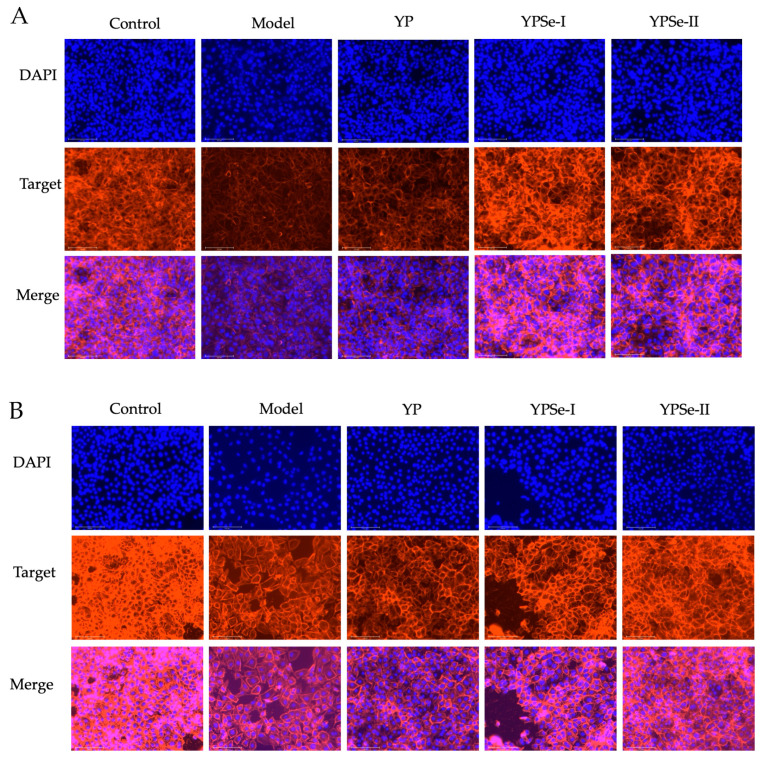
F-actin distribution of acrylamide-injured IEC-6 cells treated with three polysaccharide samples (YP, YPSe-I, and YPSe-II) at 40 µg/mL for 12 (**A**) and 24 h (**B**). The scale of the labeled bar is 125 μm.

F-actin, a key cellular protein, forms the essential cytoskeleton. Whenever the cell barrier undergoes alteration, the F-actin distribution among cells will change accordingly. Rhodamine-labeled phalloidin, with its typical affinity for F-actin, enables visualization of cytoskeletal architecture and distribution within cells. In this study, F-actin distribution in the treated cells was observed under a fluorescence microscope to verify whether they had undergone changes (Figure 7). Compared with the observation results in the control cells, the model cells exhibited weakened fluorescence signal intensity and enlarged intercellular spaces, proving the acrylamide damage to the cell barrier function. When treated with the three polysaccharide samples for 12 or 24 h, the cells were observed to have increased fluorescence signal intensity of F-actin and reduced intercellular spaces. Moreover, the effects of YPSe-I and particularly YPSe-II on F-actin distribution were more pronounced than that of YP. In other words, it was determined that YPSe-I and YPSe-II, with the covalent Se-grafting and higher selenylation extent, exhibited an enhancement in their activity in stimulating F-actin distribution and bolstering cell barrier function.

### 3.5. Effects of Three Polysaccharide Samples on Expression of Three TJ Proteins in Injured Cells

**Figure 8:**
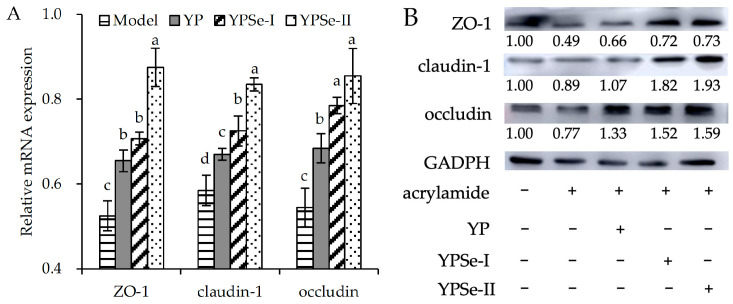
Relative mRNA (**A**) and protein (**B**) expression of tight junction proteins ZO-1, claudin-1, and occludin in acrylamide-injured IEC-6 cells treated with three polysaccharide samples (YP, YPSe-I, and YPSe-II) at 40 µg/mL for 24 h. Distinct lowercase letters above the labeled columns indicate that one-way ANOVA of the mean values differs significantly (*p* < 0.05).

In cells, TJ proteins are vital for maintaining barrier integrity. Occludin, ZO-1, and claudin-1 are all the important components of TJ proteins. In this study, the relative mRNA expression levels of the three proteins in the cells were therefore assayed. Compared with the control cells (assigned a relative mRNA expression level of 1.00-fold), the model cells exhibited a clear down-regulation in relative mRNA expression (0.52–0.58-fold) for the three proteins (Figure 8A). Regarding the model cells, the cells treated with the polysaccharide samples obtained an up-regulation in relative mRNA expression for ZO-1 (0.65–0.88-fold), claudin-1 (0.67–0.83-fold), and occludin (0.68–0.85-fold). Clearly, YPSe-I and especially YPSe-II were more effective than YP in up-regulating the mRNA expression of the three TJ proteins.

The relative protein expression levels of ZO-1, claudin-1, and occludin in the cells were also enhanced by the three polysaccharide samples (Figure 8B). Acrylamide treatment down-regulated the expression levels of the three targeted proteins (0.49–0.89-fold), relative to those of the control cells (all normalized to 1.00-fold). On the contrary, YP, YPSe-I, and YPSe-II clearly up-regulated the expression levels of ZO-1 (0.66–0.73-fold), claudin-1 (1.07–1.93-fold), and occludin (1.33–1.59-fold). Interestingly, YP and YPSe-II demonstrated the respective lowest and highest activity in up-regulating relative protein expression for the three proteins. Moreover, the change trend of protein expression was consistent with that of mRNA expression. The covalent Se-grafting enhanced the capacity of two selenylated derivatives to up-regulate the synthesis of the three TJ proteins, thus improving intestinal epithelial barrier integrity efficiently.

### 3.6. Regulation of Three Polysaccharide Samples on MAPK Signaling Pathway

**Figure 9:**
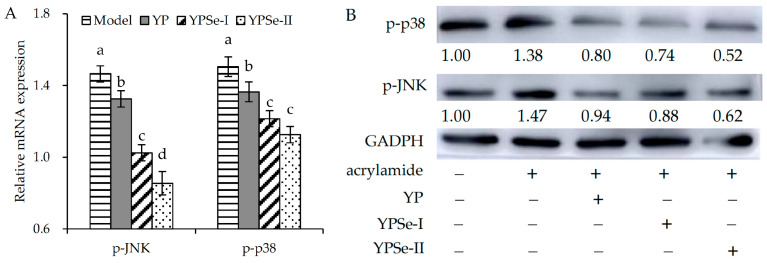
Relative mRNA (**A**) and protein (**B**) expression of p-p38 and p-JNK in acrylamide-injured IEC-6 cells treated with three polysaccharide samples (YP, YPSe-I, and YPSe-II) at 40 µg/mL for 24 h. Distinct lowercase letters above the labeled columns indicate that one-way ANOVA of the mean values differs significantly (*p* < 0.05).

Acrylamide within cells can modulate relevant signaling pathways, thus influencing cell growth or triggering cell apoptosis. The most studied signal pathways include the mitogen-activated protein kinases (MAPK) signaling pathway. Given the critical regulatory roles of p-JNK and p-p38 in activating the MAPK signaling pathway, this study implemented methodological validation through integrated RT-qPCR and Western blotting analyses to quantify their mRNA and protein expression profiles (Figure 9). Compared with the control cells, the model cells exhibited up-regulated mRNA expression for p-JNK (1.47-fold) and p-p38 (1.51-fold). When IEC-6 cells were also treated with the three polysaccharide samples for 24 h, the relative mRNA expression levels of p-JNK and p-p38 were reduced to 0.85–1.33 and 1.13–1.37-fold, respectively. Meanwhile, protein expression levels of p-JNK and p38 were elevated to 1.47 and 1.38-fold, respectively. However, the polysaccharide samples within the cells led to decreased protein expression levels of p-JNK (0.52–0.80-fold) and p-p38 (0.62–0.94-fold). These results demonstrate that acrylamide activated the MAPK signaling pathway, whereas the polysaccharide samples (particularly YPSe-II) were able to inhibit the acrylamide-induced activation of this signaling pathway. Evidently, YPSe-II showed the greatest ability in modulating p-JNK and p-p38 expression, while YP exhibited the weakest modulatory activity. It was hypothesized that the enhanced barrier repair capacity of the two Se-grafted derivatives (YPSe-I and YPSe-II) in the cells were mechanistically attributable to the obtained covalent Se-grafting and higher selenylation extent, which caused stronger inactivation of the MAPK signaling pathway and then restored the injured barrier function effectively.

## 4. Discussion

Acrylamide within the body might trigger a series of adverse events such as inducing cell damage, disrupting redox balance, and generating oxidative stress [28]. A substantial amount of past research had demonstrated the cytotoxicity of acrylamide to diverse cell types in vitro; for instance, acrylamide could induce cytotoxicity in NIH/3T3 fibroblast cells through inducing cell apoptosis [29], and might impair the morphology of A549 human lung adenocarcinoma cells [30]. Acrylamide is capable of impairing intestinal cells. In Caco-2 cells, acrylamide could lead to a significant decline in cell viability and a marked alteration in mitochondrial membrane potential, and induce oxidative stress [31]. Moreover, acrylamide can exert its cytotoxicity on non-cancer cell lines. It was reported that when 7.5 mmol/L acrylamide acted upon porcine small intestinal epithelial IPEC-J2 cells for 24 h, the cell viability dropped to approximately 60% [32]. Consistent with these reported findings, our results demonstrated that acrylamide had a toxic impact on IEC-6 cells, leading to reduced cell viability, increased LDH release, and elevated ROS production. Thus, it is necessary to verify potential harmful effects of acrylamide in the intestinal environment.

The intestine is considered to function as a principal site for nutrient digestion and absorption. Meanwhile, the intestine also serves as an innate barrier. To maintain the health of the body, the intestine exhibits a certain degree of permeability for nutrient uptake but maintains tightness to prevent the entry of external pathogens and harmful substances, thereby fulfilling its critical barrier function [33]. Natural water-soluble and non-starch polysaccharides are capable of enhancing or improving intestinal barrier function. For instance, it was verified that *Artemisia argyi* polysaccharides were capable of reducing the incidence of diarrhea in magnesium citrate-induced diarrheic rats, increasing the count of goblet cells in colonic crypts, up-regulating the expression of mucin-2 and claudin-1, thereby enhancing the intestinal barrier function [34]. It was also reported that the polysaccharides from *Armil-lariella tabescens* mycelium might alleviate dextran sulfate sodium-induced colitis symptoms in mice, because the polysaccharides were able to enhance the integrity of colon tissue by up-regulating both mucin-2 and TJ proteins as well as suppressing the MMP12/MLCK/p-MLC2 signaling pathway [35]. Additionally, *Portulaca oleracea* L. polysaccharides were reported to improve the barrier function of lipopolysaccharide-injured IPEC-J2 cells via increasing TEER, decreasing permeation of phenol red and FD-4, and up-regulating the expression of three TJ proteins including claudin-7 and ZO-1 [36]. It is well known that TJ proteins and F-actin cooperate to maintain the cell barrier function [37]. The intestinal barrier function, which is constituted by TJ proteins, regulates the permeability of intestinal substances between cells; meanwhile, F-actin ensures the effective operation of this barrier function through its supporting and stabilizing effect on TJ proteins [38]. In the presence of inflammation or stress, F-actin can modulate the state of TJ proteins through its own dynamic reorganization to sustain the barrier function between cells continuously [39]. In this study, both YP and the two Se-grafted products, when applied to IEC-6 cells, were able to enhance the TEER value, reduce paracellular permeability, enhance F-actin distribution between cells, and promote the expression of the three TJ proteins occludin, ZO-1, and claudin-1. Thus, they were considered to have the ability to counteract acrylamide-induced intestinal barrier damage.

Chemical modification of polysaccharides by the well-known carboxymethylation, acetylation, selenylation, and other approaches generally enhances their bioactivity efficiently. Carboxymethylated *Stropharia rugosoannulata* polysaccharides exhibited a better antioxidant capacity and inhibitory effect on both α-glucosidase and α-amylase [40]. After acetylation modification, *Cyperus esculentus* polysaccharides possessed higher antioxidant activity, and could alleviate the inflammatory response in LPS-induced macrophages [41]. Moreover, selenylated polysaccharides also exhibit higher bioactivity. Within the concentration range of 25–200 μg/mL, *Artemisia sphaerocephala* polysaccharides showed growth inhibition on HepG-2, Hela, and A549 cells (inhibition ratio of 12.9%); however, at 200 μg/mL, the Se-grafted *Artemisia sphaerocephala* polysaccharides showed respective inhibition ratios of 35.1%, 47.4%, and 62.2% on these cells [42]. In accordance with these results, our results also demonstrated that the Se-grafting of YP provided better protection of IEC-6 cells against acrylamide-induced cell injury, leading to reduced cell toxicity and alleviated barrier loss. Interestingly, our results also revealed that a higher selenylation extent led to an enhancement in the studied activity.

Whenever intestinal epithelial cells are damaged by external noxious substances, the intestinal barrier function can be modulated through the activation of the MAPK signaling pathway. This signaling pathway encompasses three key components, namely mitogen-activated protein (p38), extracellular regulated protein kinase (ERK), and c-Jun N-terminal kinase (JNK); moreover, it can regulate intestinal barrier function by modulating TJ proteins. External stimuli can activate p38, ERK, and JNK. The activated kinases then participate in the regulation of this signaling pathway in their phosphorylated forms [43,44,45]. Food components are capable of enhancing the intestinal barrier function through regulation of this signaling pathway. For example, it was verified by Cheng and coauthors that *Persicaria hydropiper* (L.) Spach polysaccharides modulated the intestinal barrier function in the model mice via inhibiting the phosphorylation of p38, ERK, and JNK [46]. As outlined by Cheng and coauthors, lentil hull extract (abundant in procyanidins) could enhance the barrier function of Caco-2 cells through inhibiting p-p38 expression [47]. Additionally, a milk protein, namely lactoferrin, could enhance the barrier function in model mice through regulating the MAPK signaling pathway [48]. The findings of this study confirmed that acrylamide led to an increase in p-JNK and p-p38 expression and thus triggered the MAPK signaling pathway. Conversely, YP, YPSe-I, and, most notably, YPSe-II played a role in diminishing the expression of the two proteins, thereby inhibiting the acrylamide-induced activation of the MAPK signaling pathway. It was thereby concluded that the water-soluble and non-digestive polysaccharides with covalent Se-grafting might have a higher beneficial effect in the intestinal environment, by alleviating acrylamide toxicity and restoring the acrylamide-disrupted barrier function. Moreover, this covalent Se-grafting might be useful in the modification of native polysaccharides to enhance their function in the intestine.

Collectively, it is suggested that a deeper investigation of other signaling pathways involved in the barrier protection of Se-grafted YP derivatives is also important to us. Additionally, other bioactivities of Se-grafted YP derivatives have still not been verified efficiently at the present time, especially using in vivo models. Furthermore, whether the Se-grafted YP derivatives might have different capacity in the body to regulate gut microbiota, and whether they could mitigate intestinal barrier damage caused by non-steroidal anti-inflammatory drugs, chemotherapeutics, and other medications, as well as whether they have potential as a Se supplement in Se-deficient regions, are all important research directions worthy of further exploration. It is therefore proposed that such studies are necessary and can deepen our present acknowledge of native polysaccharides and their covalent modification.

## 5. Conclusions

The soluble and nondigestive YP subjected to chemical selenylation exhibited enhanced activity in combating acrylamide-induced cytotoxicity or alleviating acrylamide-induced barrier damage in IEC-6 cells. Briefly, the selenylated products had higher Se contents, and were more capable of promoting cell viability, reducing LDH release and ROS production, increasing TEER value, reducing paracellular permeability, enhancing F-actin distribution between the cells, and up-regulating the expression of three TJ proteins. Simultaneously, the selenylated products were more effective in inhibiting the acrylamide-induced activation of the MAPK signaling pathway. Overall, chemical selenylation of YP caused covalent Se-grafting, and enhanced in vitro activity in IEC-6 cells; more importantly, higher selenylation extents yielded increased activity. The current findings also revealed that chemical selenylation can be applied to natural polysaccharides when aiming to endow them with greater potential for maintaining intestinal health. However, whether this chemical selenylation could alter other bioactivities of natural polysaccharides in the intestine also requires future studies for deeper clarification, particularly using in vivo studies.

## Figures and Tables

**Table 1:** Primer sequences for RT-qPCR analysis.

Genes	Primer Sequences (5′–3′)
ZO-1	Forward: CCACCTCGCACGTATCACAAGC
	Reverse: GGCAATGACACTCCTTCGTCTCTG
Occludin	Forward: CCTCCTTACAGGCCGGATGA
	Reverse: AGCATTGGTCGAACGTGCAT
Claudin-1	Forward: GTTTCATCCTGGCTTCGCTG
	Reverse: AGCAGTCACGATGTTGTCCC
p-p38	Forward: CCTCAGCTCAGCGAGAGAAT
	Reverse: GGCACATTTAAGCTGGGCAC
p-JNK	Forward: GCGACTGGAATGAGAACACAG
	Reverse: CTGGAACTTACTGAAGCCACC
GAPDH	Forward: CCCTCTGGAAAGCTGTGG
	Reverse: GCTTCACCACCTTCTTGATGT

## Data Availability

The original contributions presented in the study are included in the article. Further inquiries can be directed to the corresponding author.
